# Machine learning for predicting elective fertility preservation outcomes

**DOI:** 10.1038/s41598-024-60671-w

**Published:** 2024-05-02

**Authors:** Itai Braude, Einat Haikin Herzberger, Mor Semo, Kim Soifer, Nitzan Goren Gepstein, Amir Wiser, Netanella Miller

**Affiliations:** 1 Department of Obstetrics and Gynecology, Meir Medical Center, Kfar Saba, Israel; 2 School of Medicine, Tel Aviv University, Tel Aviv-Yafo, Israel; 3 OB/GYN Department, Mayanei Hayeshua Medical Center, Bnei Brak, Israel

**Keywords:** Computational models, Outcomes research

## Abstract

This retrospective study applied machine-learning models to predict treatment outcomes of women undergoing elective fertility preservation. Two-hundred-fifty women who underwent elective fertility preservation at a tertiary center, 2019–2022 were included. Primary outcome was the number of metaphase II oocytes retrieved. Outcome class was based on oocyte count (OC): Low (≤ 8), Medium (9–15) or High (≥ 16). Machine-learning models and statistical regression were used to predict outcome class, first based on pre-treatment parameters, and then using post-treatment data from ovulation-triggering day. OC was 136 Low, 80 Medium, and 34 High. Random Forest Classifier (RFC) was the most accurate model (pre-treatment receiver operating characteristic (ROC) area under the curve (AUC) was 77%, and post-treatment ROC AUC was 87%), followed by XGBoost Classifier (pre-treatment ROC AUC 74%, post-treatment ROC AUC 86%). The most important pre-treatment parameters for RFC were basal FSH (22.6%), basal LH (19.1%), AFC (18.2%), and basal estradiol (15.6%). Post-treatment parameters were estradiol levels on trigger-day (17.7%), basal FSH (11%), basal LH (9%), and AFC (8%). Machine-learning models trained with clinical data appear to predict fertility preservation treatment outcomes with relatively high accuracy.

## Introduction

The number of elective fertility preservation procedures performed have increased significantly in recent years^1^. Advances in fertility preservation treatments, coupled with the rising prevalence of delayed childbearing, a phenomenon linked to increased risk of infertility and complications during pregnancy, have led many women to choose fertility preservation as an elective procedure^2–7^.

Options for elective fertility preservation include oocyte cryopreservation, which involves extracting M2 oocytes from the ovary, and embryo cryopreservation, where the extracted oocytes are fertilized with sperm and the resulting embryos undergo cryopreservation on day 3 or 5.

Current data demonstrate that elective fertility preservation treatments are effective and outcomes after oocyte cryopreservation are comparable to those of fresh oocytes^8,9^. However, predicting treatment outcomes and the number of oocytes expected to be retrieved remains a significant challenge. This uncertainty regarding treatment success may cause patients significant psychological stress^7,10^**.**

As fertility preservation treatments have financial costs and possible adverse effects, including ovarian hyperstimulation syndrome, infection, blood loss, and anesthesia-related complications, it is important to counsel patients regarding whether the treatment is appropriate in terms of its safety, and efficacy along with their expectations^11^.

Tools have been developed to predict the likelihood of live birth after oocyte cryopreservation based on the number of M2 oocytes retrieved^12^. Yet, this study was based on regression models and did not predict the expected number of M2 oocytes.

Machine learning is a branch of computer science that focuses on data-driven computational models to make predictions based on past observations^13^. While analyzing data using standard statistical tools allows us to draw conclusions about a population from a sample, machine learning allows us to make predictions by identifying generalizable patterns in the data. We can use these predictions to determine the best course of action (e.g., treatment choice) without needing to understand the basic underlying mechanisms^13,14^.

The combination of recent developments in machine learning, along with the growing availability of medical databases, enables the medical community to build predictive models for various outcomes, which can improve customized care and resource utilization. For example, previous studies of machine learning in the field of assisted reproduction have demonstrated efficacy in predicting live births after IVF treatments^15,16^. a study that applied machine learning to estimate the number of oocytes retrieved from controlled ovarian hyperstimulation. However, it focused on couples facing fertility challenges rather than on women engaging in fertility preservation, thus examining a demographic distinct from that of our study”13).

To the best of our knowledge, there are currently no machine-learning models for predicting the success of elective fertility preservation treatments for non-medical reasons.

The objective of this study was to identify demographic, clinical, and laboratory parameters that influence treatment outcomes, and to construct a machine-learning model that will help patients and their physicians predict the number of M2 oocytes that will be retrieved during treatment. Thus, assisting physicians to better inform their patients.

## Materials and methods

### Patients

This retrospective study included 250 women, who underwent elective fertility preservation at Meir Medical Center, Israel from 2019 to 2022. Data extracted included: (1) demographics (age, BMI), (2) clinical antral follicle count (AFC), gonadotropin drugs, ovulation trigger drugs (GnRH agonist, hCG), number of stimulation days, starting dose and total gonadotropin dosage, and endometrial thickness on triggering day, (3) Laboratory values: Basal follicle-stimulating hormone (FSH), luteinizing hormone level (LH), and progesterone, estradiol, and LH levels on triggering day. At our medical center, the standard regimen for fertility preservation involves the use of an antagonist protocol, beginning with a 300IU dose of gonadotropins.

### Ethics approval

The study was approved by the Meir Medical Center Institutional Review Board (MMC-393–20). We confirm that all research was performed in accordance to declaration of Helsinki. The Institutional Review Board at Meir Medical Center, under the leadership of Prof. Ilan Cohen, approved a waiver for the requirement of informed consent.

### Outcome measures

The primary outcome was the number of metaphase II oocytes obtained during treatment. Cobo et al. suggested that at least 8 MII oocytes should be preserved to obtain a reasonable success rate of achieving a live birth (40% among women younger than 35 and 20% over the age of 35), while any additional oocytes above 15 contribute little to the cumulative live birth rate^17^.

Based on this information, we decided to categorize outcomes into three classes according to oocyte count (OC): Low (≤ 8 oocytes), Medium (9–15 oocytes), or High (≥ 16). We used different machine-learning models and logistic regression to predict the outcome class.

### Pre-treatment vs. trigger-day analysis (post-treatment)

We performed two independent analyses, training the machine-learning models on different subsets of parameters. For the pre-treatment analysis, we only used parameters obtainable during the first clinic visit, prior to initiating treatment, which included age, BMI, AFC, and basal levels of estradiol, LH, and FSH. The results of this analysis were used to evaluate potential treatment outcomes prior to initiating an IVF cycle. This may help inform the decision of whether a treatment should be started or not.

For the trigger-day analysis, we included all the parameters collected, in order to train the machine-learning models. These included all the parameters used in the pre-treatment analysis, plus estradiol, progesterone, LH, and endometrial thickness on triggering day, treatment protocols, starting dose, total dose, and the number of days of stimulation. The outcomes of this analysis were used to evaluate the efficacy of a treatment that was already started and to reduce uncertainty regarding outcomes for the patient regarding the oocyte retrieval procedure.

### Data analysis

Discrete variables are presented as numbers and percentages, and continuous variables as mean ± standard deviation (SD). We calculated p-values using student t test or χ^2^. A p-value < 0.05 was considered significant. One-way analysis of variance (ANOVA) was used to compare the means of demographic, clinical, and laboratory variables between the three outcome groups. Bonferroni correction was applied to adjust for multiple comparisons.

## Machine-learning models

### Predictor variables

Age, BMI, treatment protocols, endometrial thickness, AFC, pre-treatment levels of LH, FSH, and estradiol, and trigger-day levels of LH, estradiol, and progesterone were used as predictors. These variables are known to be related to the number of mature oocytes retrieved during fertility preservation treatments, as mentioned above.

### Model development

To assess the different machine-learning models for this task, we created an automated pipeline (steps detailed below) to pre-process the data, perform hyper-parameter tuning on each model, and compare the performance of the different models to one another. As each step involved in pre-processing the data has several options, we evaluated the models using all possible combinations of data pre-processing. To optimize the performance of the models, each was tested with 100 different combinations of parameters that affect how the model performs. Each model was evaluated 3 times using threefold cross-validation, where the data are split into 3 parts, 2 for training and 1 for validation.

The scoring function calculated the area under the curve (AUC) for the receiver operating characteristic (ROC) curve for each class against all others. The ROC curve is a graphical plot that demonstrates the performance of binary classifiers by plotting the true positive rate as a function of the false-positive rate. In this multi-class setting, ROC curves can be extended by considering each class as a binary classification (one class versus all other classes), plotting a ROC curve for each class. The AUC for each class is then computed, and these can be averaged in various ways, such as micro-averaging, which aggregates the contributions of all classes to compute the average AUC, or macro-averaging, which computes the AUC independently for each class and then takes the average. Higher average AUC ROC scores indicate more accurate multi-class models.

The pipeline had the following steps:

1. The dataset was split, using 70% for training the models and saving 30% for the test set.
2. Data imputation. Four different options were tested, including constant, mean, median, and most frequent value imputation.
3. Feature scaling was used to avoid model bias due to differences in variable scales. We tested standard scaling, min–max scaling, and no scaling.
4. Feature reduction was tested using principal component analysis compared to no feature reduction.
5. For each of the possible combinations of steps 2–4, the following classifiers were tested: Logistic Regression, Support Vector Machine, Random Forest, XGBoost, Naïve Bayes, and K-Nearest Neighbours. Each model underwent hyper-parameter tuning using a random grid search algorithm and threefold cross-validation. Each model reported the mean ROC AUC score and standard deviation.

Upon completion of the grid search, the parameters that yielded superior performance were selected for our data pre-processing strategy. Mean imputation was employed to address missing values due to its consistent effectiveness across our dataset. Min–max scaling was chosen to normalize feature scales, which contributed to improved model predictive performance. Feature reduction was not implemented, as the grid search indicated that the use of the full dataset maximized the informative value of the models.

### Model selection

The models were ranked using the following formula:

$$rank=mean-2\times SD$$

The models with the highest rankings were selected and used to predict the results of the test set.

All analyses were done using Python v2.7.16 and Sci-kit Learn, version 1.1.0.

## Results

A total of 250 women who underwent elective fertility preservation and were treated with an antagonist protocol were included. According to the number of oocytes retrieved, 136 were in the Low OC class (≤ 8 or fewer oocytes), 80 were in the Medium OC class (9–15), and 34 were in the High OC class (≥ 16). The average age of the entire cohort was 35 years, average BMI was 23, average AFC was 14, and the average basal FSH was 8.5.

### Comparison between women with Low, Medium or High OC outcomes

Age, BMI, basal estradiol levels, and LH levels on triggering day did not differ significantly between groups (Table 1). The difference in basal LH levels between the Low and Medium OC groups almost reached statistical significance (p = 0.087).

**Table 1:** Demographic, clinical and laboratory data for patients with outcomes classified as low oocyte count vs. patients with outcomes classified as medium or high oocyte count.

Characteristic	Outcome Class			p-value		
	Low OC (n = 137)	Medium OC (n = 80)	High OC (n = 34)	Low vs. Medium	Low vs. High	Medium vs. High
Demographics						
Age, years ± SD	35.3 ± 2.9	35.2 ± 2.1	34.5 ± 1.9	1.00	0.358	0.630
Body mass index	22.7 ± 4.1	23.3 ± 4.4	23.7 ± 4.2	1.00	0.690	1.00
Clinical and laboratory data						
Antral follicle count	12.2 ± 5.2	16.5 ± 8.2	16.7 ± 9.7	0.000	0.002	1.00
E2, basal	55.86 ± 25.14	65.98 ± 92.88	84.41 ± 139.82	1.00	0.149	.703
LH, basal	5.37 ± 3.35	6.34 ± 2.63	6.44 ± 3.14	0.087	0.221	1.00
FSH—basal	9.32 ± 3.75	7.79 ± 2.08	6.75 ± 1.69	0.001	0.000	0.299
E2 triggering day	1982.1 ± 1073.9	3295.1 ± 1531.6	4749.5 ± 2029.4	0.000	0.000	0.000
LH triggering day	4 ± 8.2	3.46 ± 11.12	1.63 ± 1.01	1.00	0.473	0.925

*FSH* follicle stimulating hormone, *LH* luteinizing hormone, *OC* oocyte count, *SD* standard deviation.

Women with Low OC had significantly lower AFC (12.2 ± 5.2 vs. 16.5 ± 8.2, p = 0.000 and 12.2 ± 5.2 vs. 16.7 ± 9.7, p = 0.002) and significantly higher basal FSH levels (9.32 ± 3.75 vs. 7.79 ± 2.08, p = 0.001 and 9.32 ± 3.75 vs. 6.75 ± 1.69, p = 0.00), respectively, compared to the Medium and High OC groups. On triggering day, the Low OC group had significantly lower estradiol levels and the High OC group had significantly higher levels (1982.1 ± 1073.9 vs. 3295.1 ± 1531.6 vs. 4749.5 ± 2029.4, p = 0.000; Table 1).

### Pre-treatment machine-learning model

**Figure 1a:**
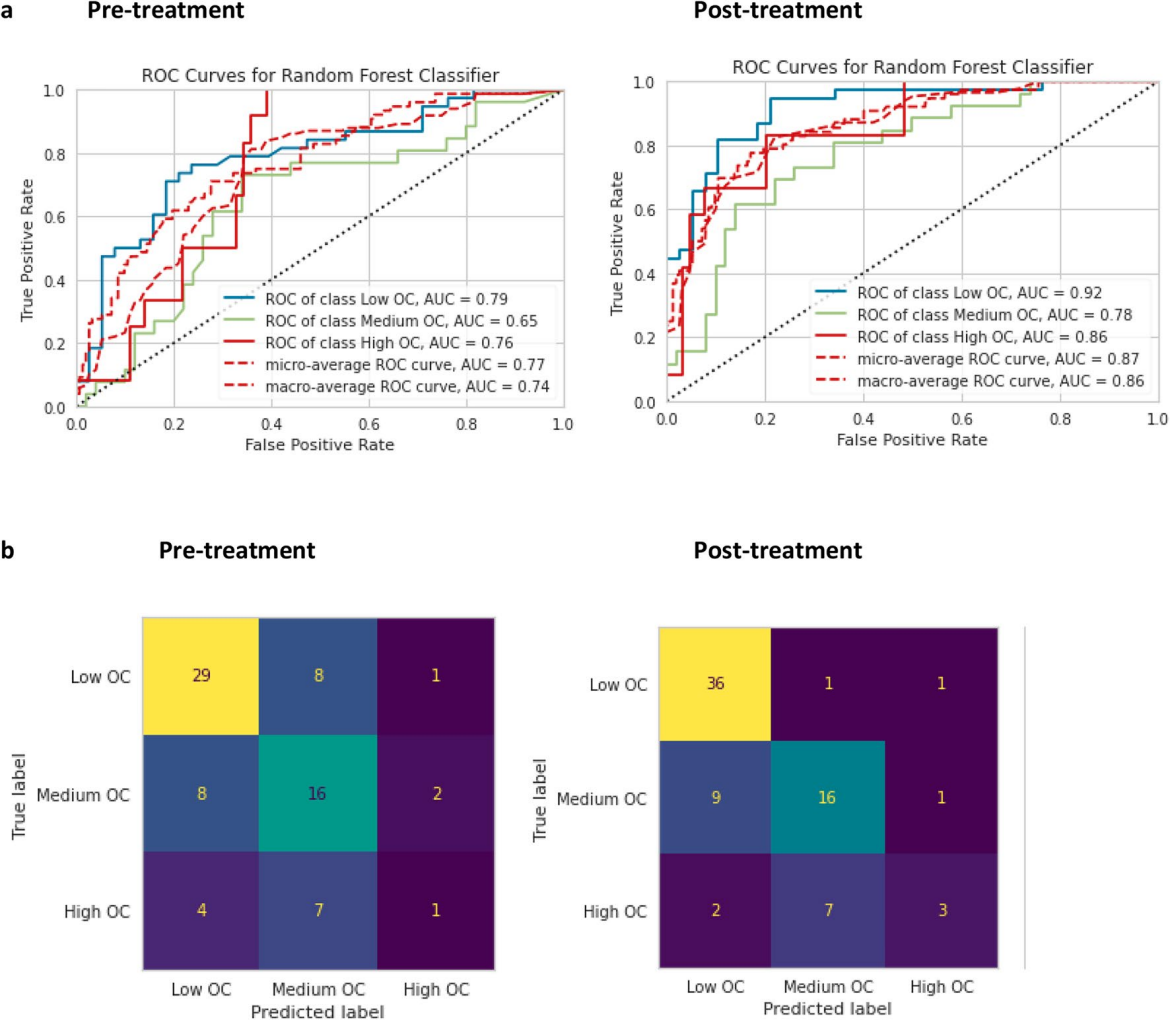
Pre-treatment and post-treatment receiver operating characteristic (ROC) curves for the Random Forest Classifier comparing ROC AUC for the different outcome classes. Figure 1**b**. Confusion matrix for the pre-treatment and post-treatment for Random Forest Classifier.

**Figure 2a:**
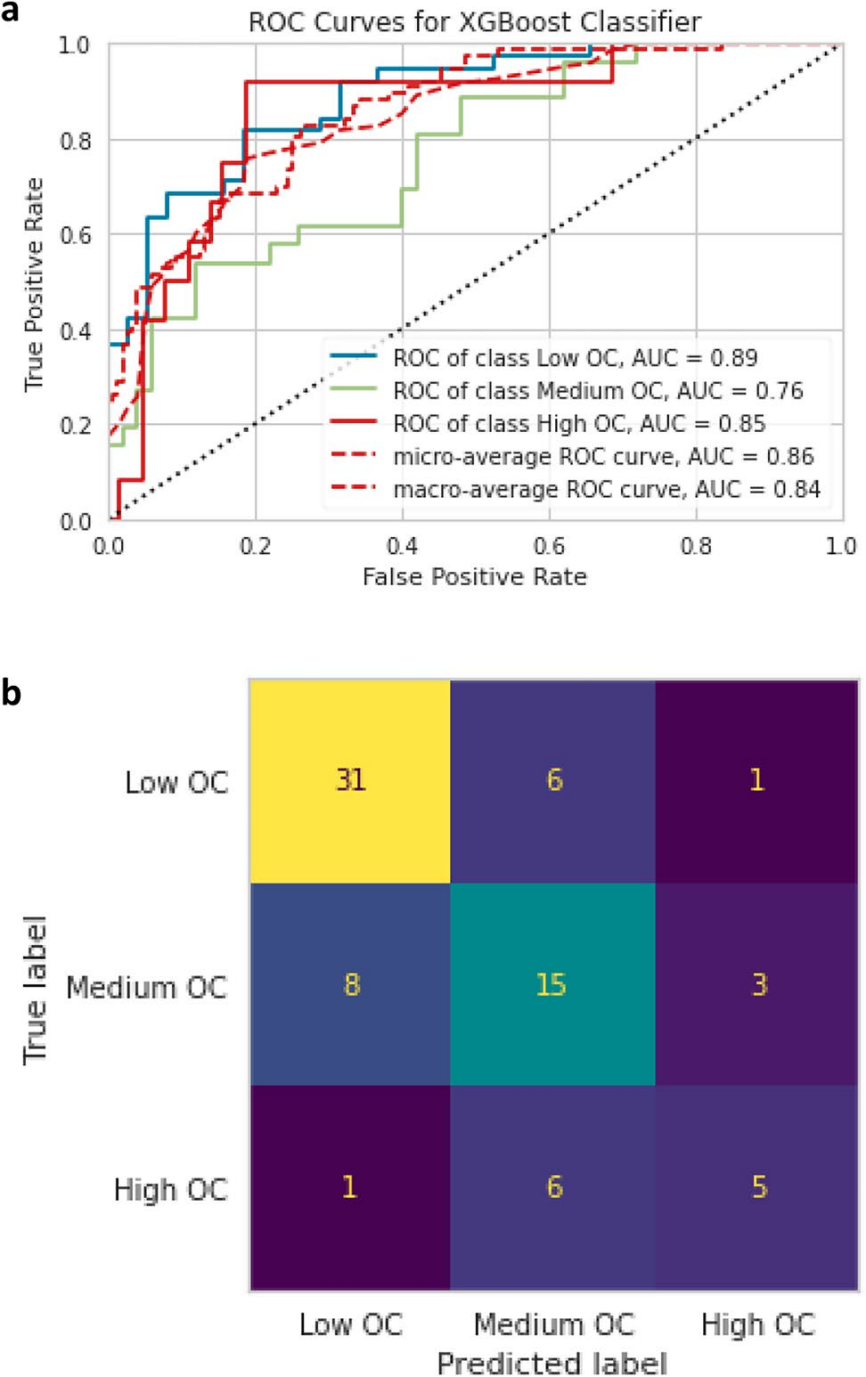
Post-treatment ROC curves comparing ROC AUC for the outcome classes of the XGBoost Classifier. Figure 2**b** Post-treatment confusion matrix for the XGBoost Classifier.

For the pre-treatment analysis, the Random Forest Classifier was the most accurate model (ROC AUC 77%; Fig. 1a), followed by the XGBoost Classifier (ROC AUC 74%; Fig. 2b), and then by the Logistic Regression Classifier (ROC AUC 68%). The least accurate model was the Naïve Bayes Classifier (ROC AUC 59%).

**Figure 3a:**
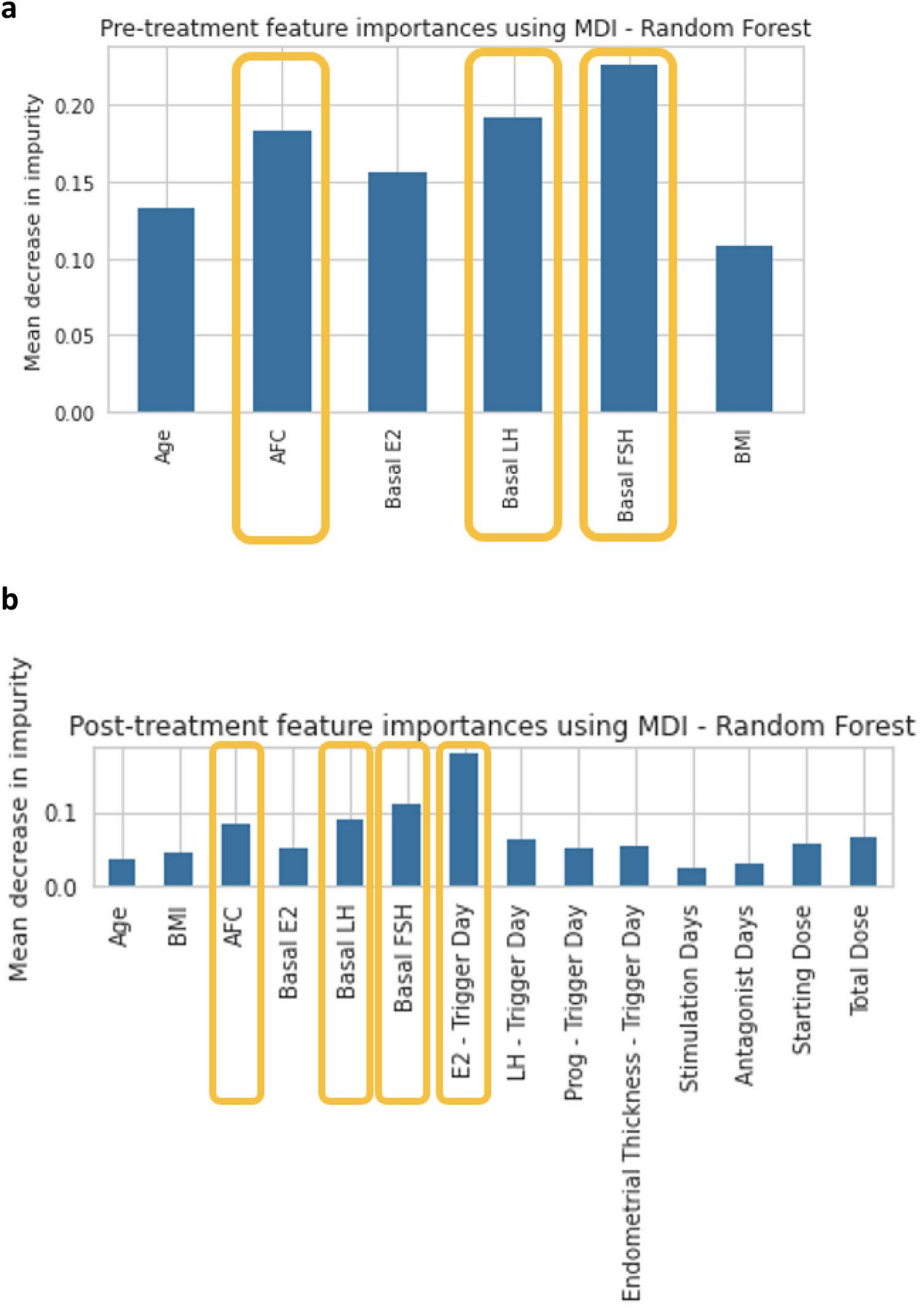
Important features pre-treatment, using mean decrease in impurity—Random Forest Classifier. Figure 3**b** Important features post-treatment, using mean decrease in impurity—Random Forest Classifier.

The sensitivity and specificity for the Random Forest Classifier (Table 2, Fig. 1b) were 76.3% and 68.4% for the Low OC class, 61.5% and 70% for the Medium OC class, and 8.3% and 95.3% for the High OC Class. The best predictive parameters for the Random Forest Classifier (Fig. 3a) were basal FSH (importance 22.6%), basal LH (importance 19.1%), AFC (importance 18.2%), and basal estradiol (15.6%).

**Table 2:** Specificity and sensitivity of the pre-treatment and post-treatment for Random Forest Classifier, according to oocyte count outcome class.

Pre-treatment	Sensitivity	Specificity
Low OC	76.3%	68.4%
Medium OC	61.5%	70%
High OC	8.3%	95.3%
Post-treatment		
Low OC	94.7%	71%
Medium OC	61.5%	84%
High OC	25%	96.8%

*OC* oocyte count.

### Post-treatment machine-learning model

For the post-treatment analysis, the most accurate classifier model was the Random Forest Classifier (ROC AUC 87%; Fig. 1A), followed by the XGBoost Classifier (ROC AUC 86%; Fig. 2a, and by the Logistic Regression Classifier (ROC AUC 84%). The least accurate model was the Naïve Bayes Classifier (ROC AUC 64%).

The sensitivity and specificity for the Random Forest Classifier (Table 2, Fig. 2b) were 94.7% and 71% for the Low OC class, 61.5% and 84% for the Medium OC class, and 25% and 96.8% for the High OC Class. The XGBoost model had higher specificity for the Low OC Class (sensitivity 81.5%, specificity 76.3%) and was more sensitive for the High OC Class (sensitivity 41.6% and specificity 93.7%;). The Random Forest Classifier (Fig. 3b) had the best predictive parameters for estradiol levels on trigger-day (importance 17.7%), basal FSH (importance 11%), basal LH (importance 9%), and AFC (importance 8%).

## Discussion

The present study compared the ability of different machine-learning models to accurately predict the outcomes of elective fertility preservation treatments according to the women’s demographic and clinical data. Based on the number of metaphase II oocytes obtained during treatment, we defined three outcome classes that correlated with the success rate of the treatment ultimately resulting in mature oocytes, and used machine-learning models to predict a woman’s outcome class at two points: before starting treatment and post-treatment on ovulation triggering day. We demonstrated that using machine learning techniques, we were able to predict the number of mature oocytes with superior sensitivity and specificity compared to logistic regression analysis.

The Random Forest Classifier model performed best for both pre-treatment (ROC AUC 77%) and trigger-day analyses (ROC AUC 87%). In terms of predictive values, these measures are considered high; demonstrating the feasibility of using the model in clinical practice. The highest sensitivity and specificity for identifying the low OC class (≤ 8 oocytes) were found in the pre-treatment analysis (76.3 and 68.4%, respectively), with even higher results in the post-treatment analysis (94.7 and 71%).

As the treatment itself provides information about the ovarian response of a specific patient, it is not surprising that the model had a higher degree of sensitivity and specificity in the post-treatment analysis.

Even though the pre-treatment model was less accurate than the post-treatment model, it provided a sufficiently high degree of predictive performance even before starting treatment, which suggests that it may be a clinically effective tool for advising patients and setting realistic expectations regarding fertility preservation treatments. This is especially important due to the risk of adverse effects, financial costs, and the emotional burden associated with these procedures.

The variables with the highest predictability for both post-treatment and pre-treatment models included basal FSH, basal LH and AFC. The post-treatment model also included estradiol levels as the most important feature.

While it is known from previous studies that FSH, AFC, and estradiol levels correlate with ovarian response^18,19^, few studies have addressed LH levels as a predictor of ovarian response. The current study may shed light on the importance of considering LH levels when consulting patients regarding ovarian reserves. Further studies are required to substantiate this finding.

In our models, age and BMI were minimally important as predictors for the number of M2 oocytes retrieved during treatment, even though a patient’s age is known to be a significant factor when assessing ovarian reserves and the likelihood of achieving a live birth^17,20^. A possible explanation is that the ages of the groups did not vary significantly, which led to a weaker predictive value for this variable.

The importance of this study is that it demonstrates the ability to provide clinicians with tools powered by machine-learning models to predict treatment outcomes when considering fertility preservation treatments for patients, especially when expectations regarding treatment outcomes are low.

This study had some limitations. First, anti-Mullerian hormone (AMH) measurements were not used as a parameter despite the strong correlation between AMH serum levels and ovarian reserves^21^. As testing AMH serum levels require an additional out-of-pocket payment in Israel, many patients choose to forgo the test. As more than half of our patients were missing AMH measurements, and considering that AFC measurements are equally accurate in assessing ovarian reserves^19^, we decided to omit AMH from the model. Second, the predictive power of machine-learning classifiers strongly depends on the quality and size of the training data^22^. The relatively small sample size in this study may underestimate the possible predictive performance and power of these models. Third, the three outcome groups in our study were not similar in size., The High OC class had 34 patients compared to the Low OC class with 136 patients. This may have contributed to the low sensitivity of the models in predicting the High OC class.

Despite the shortcomings of this study, we believe that it serves as a good proof-of-concept for the utilization of machine learning models as part of the shared decision-making process of physicians and patients regarding elective fertility preservation treatments. In addition, both pre- and post-treatment models had the highest sensitivity and specificity for predicting the Low OC outcome class. We believe that predicting poor treatment outcomes would be the most useful clinically, as it could allow patients with poor chances of success to make an informed decision before starting treatments or undergoing oocyte retrieval. Further model training on larger datasets is required to improve the model’s predictive performance and power. Extensive clinical validation of these models is also needed before such a model could be used in clinical practice. Additional studies with larger sample sizes are required to confirm our findings.
